# Artificial intelligence in corneal endothelial disease and endothelial keratoplasty: current evidence, clinical readiness, and future directions

**DOI:** 10.3389/fopht.2026.1951354

**Published:** 2026-09-15

**Authors:** Anđela Jukić, Kata Čulina, Vedran Markušić, Sanja Masnec, Suzana Matić, Miro Kalauz

**Affiliations:** 1 Department of Ophthalmology, Clinical Hospital Dubrava, Zagreb, Croatia; 2 Department of Ophthalmology, Zadar General Hospital, Zadar, Croatia; 3 Department of Ophthalmology, University Hospital Centre Zagreb, Zagreb, Croatia; 4 School of Medicine, University of Zagreb, Zagreb, Croatia; 5 Department of Ophthalmology, University Hospital Osijek, Osijek, Croatia; 6 Department of Ophthalmology and Optometry, Faculty of Medicine Osijek, Josip Juraj Strossmayer University of Osijek, Osijek, Croatia

**Keywords:** anterior-segment optical coherence tomography, artificial intelligence, clinical translation, corneal endothelium, DMEK, endothelial keratoplasty, Fuchs endothelial corneal dystrophy, specular microscopy

## Abstract

Corneal endothelial disease generates high-dimensional imaging and longitudinal data, making it well suited to artificial intelligence (AI). This review follows AI across the endothelial care pathway and judges each application by translational maturity rather than discrimination alone, drawing on a targeted PubMed/MEDLINE search of studies published between 2011 and July 2026. Technical performance is strongest when the task is measurement against a reproducible human reference: endothelial cell segmentation, image quality control, edema and pachymetric mapping, and quantification of Descemet membrane endothelial keratoplasty (DMEK) detachment. However, performance declines as tasks become prognostic or when models cross devices, surgeons, and centers. External validation reduced the area under the curve from 0.96 to 0.77 in one Fuchs endothelial corneal dystrophy (FECD) detection model, and a 2025 systematic review identified external validation in only three of 19 FECD studies. Calibration, uncertainty quantification, decision curve analysis, and prospective human–AI comparison remain uncommon. The most mature applications are therefore measurement and quality-control tasks, including automated donor endothelial cell density assessment in eye banking. FECD staging, prediction of decompensation after cataract surgery, the need for rebubbling (reinjection of air or gas into the anterior chamber to promote graft reattachment), rejection, and long-term graft survival remain investigational. By covering the full clinical pathway together with eye banking, regenerative therapies, foundation models, language models, an explicit clinical-readiness framework, and the European regulatory context, this review outlines the current requirements for clinical translation: multicenter external validation, transparent reporting, safe abstention, and prospective clinical impact studies.

## 1 Introduction

The corneal endothelium is a monolayer that, *in vivo*, essentially does not divide. It maintains stromal deturgescence through combined barrier and pump function, drawing on a cell reserve that only declines. As cells are lost and the remaining cells enlarge and lose hexagonal regularity, the cornea begins to swell. Patients report blurred vision that is worst upon waking, glare, and a loss of contrast sensitivity that is poorly reflected by visual acuity; in advanced disease, ruptured bullae cause pain. Fuchs endothelial corneal dystrophy (FECD) and pseudophakic bullous keratopathy account for most endothelial failure cases seen in practice (1, 2).

Endothelial keratoplasty changed what can be offered to these patients. Descemet stripping automated endothelial keratoplasty (DSAEK) and, increasingly, Descemet membrane endothelial keratoplasty (DMEK) replace only the diseased posterior layers, avoiding much of the optical and structural cost of penetrating keratoplasty (3, 4). What surgery failed to resolve is the uncertainty surrounding each decision. Should a patient with FECD and a visually significant cataract undergo phacoemulsification alone, combined surgery, or staged keratoplasty? Does a given detachment require rebubbling? Which grafts warrant closer surveillance for rejection or failure? Experienced surgeons draw different conclusions from the same findings.

Artificial intelligence (AI) is attractive here because a corneal consultation already means weighing symptoms against visual acuity, slit-lamp appearance, pachymetry, specular microscopy, anterior-segment optical coherence tomography (AS-OCT), and Scheimpflug tomography and, after transplantation, donor characteristics and a long series of postoperative measurements. Conventional machine learning handles non-linear relationships in structured data of this kind; deep learning can work directly with the images themselves.

The literature has grown quickly but unevenly. A 2025 systematic review of AI in FECD identified 19 studies, of which only three had been externally validated (5). Broader reviews have surveyed the use of AI across corneal and ocular surface diseases (6–8). We take a different route, following the endothelial patient along the entire clinical pathway: quantitative imaging and FECD phenotyping, decision-making during cataract surgery, follow-up after keratoplasty, long-term graft survival, eye banking, and emerging cell-based therapies. For each application, we ask three questions rather than one: How well was it validated? Is it ready for clinical use? Would it change management?

## 2 Methods: scope, literature identification, and evidence appraisal

This article is a structured narrative review rather than a systematic review. PubMed/MEDLINE was searched using targeted combinations of the following concepts: artificial intelligence, machine learning, deep learning, neural networks, random forests, computer vision, and automated image analysis; together with corneal endothelium, endothelial cell density, specular microscopy, confocal microscopy, anterior-segment optical coherence tomography, Scheimpflug tomography, Fuchs endothelial corneal dystrophy, corneal edema, DMEK, DSAEK, endothelial keratoplasty, graft detachment, rebubbling, rejection, graft failure, donor cornea, and eye bank. Additional iterative searches addressed cultured corneal endothelial cells, corneal endothelial cell therapy, regenerative therapy, induced pluripotent stem cells, foundation models, and language models to provide clinical and technological context. Reference-list screening and forward citation tracking were used to identify additional primary studies and relevant reviews. The search covered publications from January 2011 to July 2026 and was last updated in July 2026. The search was deliberately targeted and iterative, consistent with a narrative review, and was intended to support an interpretive clinical synthesis rather than exhaustive evidence enumeration. Conventional automated image-analysis systems were included when they addressed the same clinically relevant measurement tasks as AI models and provided an informative benchmark for clinical translation.

Records identified through the targeted searches were screened iteratively for clinical relevance at title/abstract level, followed by full-text assessment of potentially eligible studies. Eligible primary studies were English-language human studies that directly evaluated AI, machine-learning, deep-learning, or automated image-analysis approaches relevant to corneal endothelial disease, endothelial keratoplasty, or donor tissue assessment and reported sufficient information on the dataset, independent units of analysis, reference standard, validation strategy, and a clinically relevant quantitative, diagnostic, prognostic, or workflow outcome. Studies that focused exclusively on non-endothelial corneal disease, non-human models, or purely technical methods without a clinically interpretable evaluation were not retained for the primary evidence synthesis. Seminal clinical studies published before 2015 were retained when required for biological or surgical context. Because the aim was narrative clinical synthesis rather than exhaustive systematic review, no Preferred Reporting Items for Systematic Reviews and Meta-Analyses (PRISMA) flow diagram, protocol registration, duplicate screening, formal risk-of-bias instrument, or meta-analysis was undertaken. Quantitative pooling was considered inappropriate because studies differed substantially in disease spectrum, image acquisition, units of analysis, model architecture, outcome definitions, and validation strategy. Among the cited evidence, 23 primary studies directly evaluated AI or automated image-analysis systems in corneal endothelial disease, keratoplasty, or eye banking. These studies were published between 2019 and 2026, with additional contextual clinical studies dating from 2011 onward. The synthesis therefore emphasizes representative primary studies, external validation, calibration, and clinical applicability rather than pooled accuracy.

For the purposes of this narrative review, we developed an author-defined qualitative framework for translational appraisal and classified applications as conceptual, exploratory, early, early-to-moderate, or moderate. Ratings were applied at the application level and considered four domains: independence and diversity of validation; clinical relevance of the endpoint; reporting of calibration, uncertainty, and failure modes; and evidence of prospective workflow or patient-level benefit. “Conceptual” denoted a clinically plausible use case without endothelial-specific model validation. “Exploratory” denoted proof-of-concept evidence from small or single-center datasets with internal validation only. “Early” denoted patient-level evaluation without reliable external validation or clinical-impact evidence. “Early-to-moderate” denoted a clinically relevant endpoint with at least one independent validation, temporal evaluation, or cross-site transfer assessment. “Moderate” denoted repeated validation of a measurement or workflow task with clinically interpretable error, but did not imply regulatory authorization, autonomous use, or demonstrated improvement in patient outcomes. All authors reviewed the evidence and reached consensus on the ratings. These categories represent an interpretive clinical synthesis rather than a formal evidence-grading system.

## 3 Clinical tasks and the appropriate role of AI

Corneal endothelial disease is well-suited for AI because its clinical pathway relies on repeated quantitative measurements and images that are difficult to interpret. However, it is important to distinguish the tasks AI is being asked to perform. Some systems measure by identifying cell borders, guttae, corneal interfaces, edema, or the margin of a detached graft. Others diagnose by distinguishing FECD from other corneal states and estimating the stage. A third group is prognostic, estimating the probability of keratoplasty, rebubbling, rejection, or graft failure. A fourth manages workflow by discarding ungradable images, prioritizing high-risk examinations, and standardizing follow-up reports (5–8). **Figure 1** shows these task types across the endothelial care pathway.

**Figure 1:**
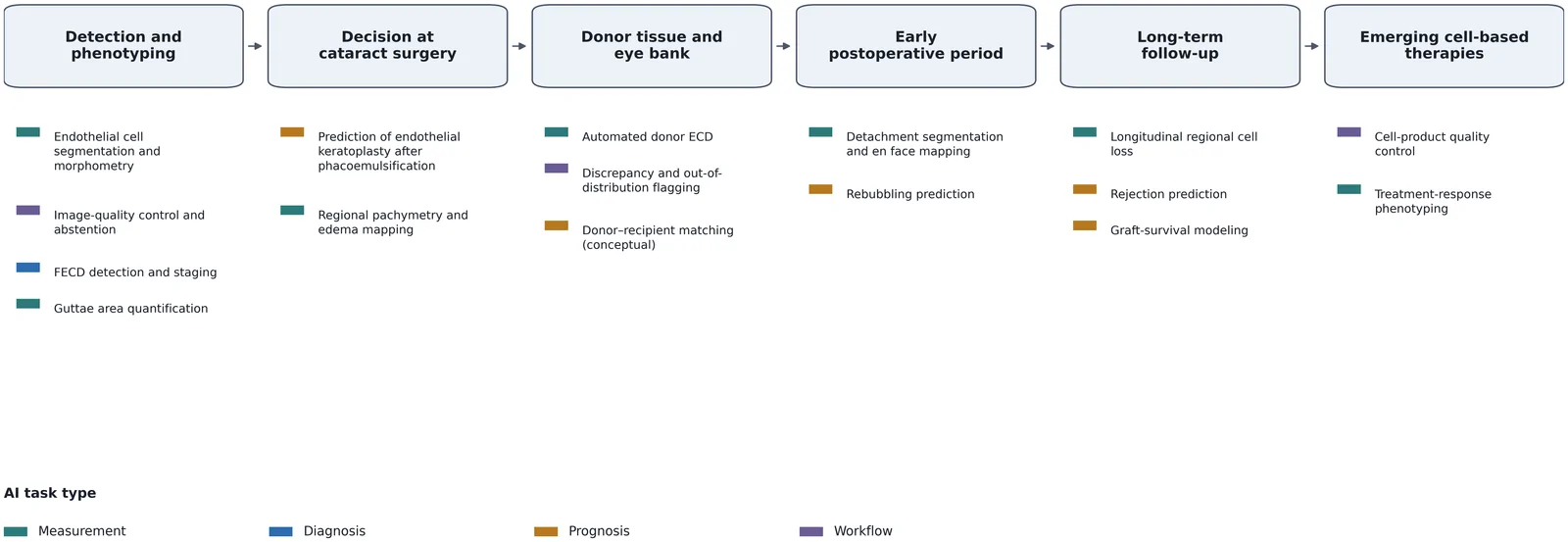
Artificial intelligence tasks across the corneal endothelial care pathway. Clinical stages are shown along the top row. Beneath each stage are the artificial intelligence applications discussed in this review, color-coded by task type: measurement, diagnosis, prognosis, and workflow. Task types are not interchangeable and require different forms of evidence before clinical use. AS-OCT, anterior-segment optical coherence tomography; DMEK, Descemet membrane endothelial keratoplasty; ECD, endothelial cell density; FECD, Fuchs endothelial corneal dystrophy; LLM, large language model.

Conflating these categories is the most common error when reading this literature. Accurate segmentation is a measurement tool; it proves nothing about patient outcomes. A high diagnostic area under the receiver operating characteristic curve (AUC) does not indicate whether a treatment decision improved patient outcomes. Readiness for clinical use means that the intended use, target population, time horizon, comparator, and management consequence were defined before the study began, not inferred afterward. The evidence needed to justify a model rejecting poor-quality scans differs from that needed to recommend combining cataract surgery with DMEK.

Endothelial datasets present characteristic challenges. Images from the same patient, or both eyes of one patient, are easily split across training and test partitions, and the resulting leakage inflates performance. Furthermore, cohorts assembled in tertiary practice over-represent severe disease, which flatters discrimination performance relative to routine practice. A model trained on one device may be learning that manufacturer’s rendering style rather than the underlying biology of the disease. Treatment itself alters the outcome being modeled: performing a combined DMEK prevents the post-phacoemulsification decompensation that the model is designed to predict. These are design problems first and interpretation problems second.

Reported performance metrics should not be interpreted as directly comparable across studies. Cohorts differ substantially in disease spectrum and severity, imaging devices and acquisition protocols, units of analysis, reference standards, outcome definitions, and validation strategies. Accordingly, AUCs, sensitivities, specificities, Dice scores, and related metrics are presented to characterize performance within individual studies rather than to rank algorithms across studies.

## 4 Quantitative imaging and FECD phenotyping

### 4.1 Specular and confocal microscopy segmentation

Automated endothelial analysis is the ideal starting point, as manual cell border correction is time-consuming, and the software supplied with the instruments performs poorly on low-contrast, postoperative, and guttae-rich images. Applied to a large real-world specular microscopy dataset, a U-Net segmentation approach correlated strongly with manual endothelial cell density (ECD), yet cell-level precision and recall exposed important failure modes and highlighted the need for explicit flagging of ungradable images (9). Image-level correlation is therefore not sufficient: an algorithm can approximate the correct average ECD while misplacing individual cell boundaries.

Guttae produce dark, confluent regions that cause generic segmentation to fail, so disease-specific methods have been built for them. Sierra et al. reframed the task as a signed distance map regression, achieving results that closely match manual FECD morphometry. Their method showed a mean ECD difference of −41.9 cells/mm² and a mean cell area difference of 14.8 µm² (10). Evaluating agreement through these metrics is clinically superior to using pixel accuracy, as clinical decisions are driven by ECD, coefficient of variation, hexagonality, cell area distribution, and guttae burden.

A prospective 2026 study took a different approach, validating an automated guttae area ratio (GAR) against expert manual grading. Manual and automated GARs were found to be equivalent in the 19-image validation set, and in an additional 446 images from 109 eyes, GAR increased alongside the modified Krachmer grade, showing high repeatability (intraclass correlation coefficient 0.85) (11). The platform uses conventional automated image processing rather than deep learning, which is arguably its strength, as longitudinal monitoring and therapeutic trials require an interpretable, repeatable phenotype.

Post-keratoplasty images are even more demanding. A deep-learning system applied to 383 images from 41 eyes after ultrathin DSAEK returned ECD, coefficient of variation, and hexagonality in 98.4% of images, against 71.5% for ECD and coefficient of variation and only 30.5% for hexagonality using the device software; the mean percentage errors were 2.5%, 5.7%, and 5.7%, respectively (12). A later model analyzing 506 postoperative images reached an accuracy of 86.2%, an F1 score of 0.857, and an AUC of 0.764 for cell detection and segmentation (13). Automated morphometry clearly works in this setting, but remains dependent on device, imaging protocol, and annotation quality.

In routine use, such software should report the analyzed area, the number of accepted cells, a quality score, an estimate of uncertainty, and the reason for any analysis failure. It should distinguish true guttae from shadowing and allow targeted manual correction. In advanced FECD, reliable abstention is more valuable than a precise but misleading ECD estimate.

### 4.2 AS-OCT pachymetry and edema mapping

AS-OCT adds cross-sectional and volumetric information: corneal thickness, interface irregularity, hyperreflectivity, and edema. Delineating these boundaries automatically is difficult in irregular and postoperative corneas. Heslinga et al. trained three deep-learning strategies on 960 B-scans from 50 post-DMEK patients and tested them on an independent set of 320 B-scans, achieving a central 9-mm pachymetry error of 14.0–15.5 µm (under 3% of average corneal thickness) that was precise enough to support regional and differential pachymetric maps (14). The clinical value lies in longitudinal registration, as the location of corneal swelling may matter more than a single central number.

Bitton et al. classified pixels on corneal OCT images as normal or edematous across 1,992 images from 290 eyes. Using postoperative reduction in central corneal thickness as the reference, the edema fraction reached an AUC of 0.97 overall and 0.96 when analysis was restricted to FECD and normal eyes (15). Heat maps make focal edema visible in a way central pachymetry cannot. The endpoint, however, was detection of edema before DMEK, not whether vision or surgical outcome would improve.

### 4.3 FECD diagnosis, staging, and widefield assessment

Deep learning can also classify FECD from AS-OCT and specular microscopy. Eleiwa et al. trained a model on 11,340 AS-OCT images and evaluated 7,380 unseen scans from 41 eyes, reporting an AUC of 0.997 for early and 0.974 for late FECD. The model achieved a sensitivity and specificity of 91% and 97% for early disease, and up to 100% and 92% for late disease, respectively (16). Technically, this is convincing. Forty-one independent eyes, all imaged in one clinical environment, are nevertheless a narrower base than the image counts suggest.

Widefield specular microscopy may capture the patchy distribution of surviving endothelium better than central imaging. In a pilot study of 775 central images with an external set of 180, internal FECD detection was strong (AUC 0.96, sensitivity 91%, and specificity 91%), but external performance fell to an AUC of 0.77 with a sensitivity of 69% and a specificity of 68% (17). That decline is among the more instructive findings in this literature, as it quantifies the distance between internal cross-validation and real-world generalization. A second model in the same work identified peripheral images with ECD above 1,000 cells/mm² at an AUC of 0.88, a task that becomes relevant once Descemet stripping only or cell-based therapy is under consideration (17).

More recent widefield work used 1,839 images from 155 FECD eyes and a separate training cohort of FECD and control eyes. A U-Net-based model reached a mean Dice coefficient of 0.86, yet automated central ECD was substantially higher than that of manual analysis (18)—excellent segmentation overlap and interchangeable clinical measurement are distinct achievements. Deep learning-assisted widefield imaging after DMEK has also been used to classify image quality, low ECD, and longitudinal regional cell loss, with AUC values of approximately 0.91 to 0.98 (19). Such tools can standardize follow-up provided the thresholds are prespecified and linked to clinical action.

## 5 Predicting endothelial decompensation after cataract surgery

In patients with FECD and cataracts, the question is not simply whether FECD is present, but whether phacoemulsification alone will keep the cornea clear. We can now find the answer by combining symptoms, slit-lamp findings, ECD (where it can still be measured), central corneal thickness, posterior corneal backscatter, irregular isopachs, displacement of the thinnest point, posterior elevation, cataract density, and clinical experience (20–23). No single threshold among these factors is universally reliable, and patients and surgeons weigh the trade-offs differently.

A 2026 multicenter study addressed this question directly using Scheimpflug tomography and a hybrid deep-learning framework combining self-supervised pretraining, ensemble learning, and clinically informed spatial processing. The labeled cohort comprised 240 eyes from 211 patients across five hospitals. The model predicted the need for endothelial keratoplasty after cataract surgery with an overall AUC of 0.94, outperforming biomarker-based approaches, and a leave-one-center-out evaluation still yielded a mean AUC of approximately 0.91 (24). Two features make this an advance: the endpoint is clinically meaningful, and generalization was tested across hospitals rather than just assumed.

However, retrospectively predicting who underwent keratoplasty is not the same as predicting who needed it. The observed endpoint incorporates referral patterns, local thresholds, access to surgery, patient preference, and surgeon judgment, alongside biological decompensation. Before clinical use, such a model should be evaluated in temporally and geographically independent cohorts, calibrated to absolute risk over a stated interval, and benchmarked against a transparent clinical rule. The decisive study would be prospective: Does model-supported counseling reduce unnecessary combined procedures without delaying treatment for eyes that decompensate?

The longer-term aim is causal rather than prognostic: to estimate what would happen to the same patient under phacoemulsification alone, combined DMEK, and staged surgery. Because the treatment chosen determines the observed outcome, ordinary supervised learning risks replicating current practices instead of improving them. Target-trial emulation, causal inference, and decision curve analysis offer better solutions.

## 6 AI in DMEK planning and early postoperative management

### 6.1 Prediction of detachment and rebubbling

Partial graft detachment is the most common early complication after DMEK, and the threshold for rebubbling varies with detachment size, central involvement, edema, the time since surgery, and surgeon preference. Patefield et al. applied multiple-instance learning to 9,466 preoperative AS-OCT images from 74 eyes. In the held-out test cohort, the sensitivity was 92%, specificity was 45%, and AUC was 0.63 for clinically significant detachment requiring rebubbling (25). A sensitivity of this magnitude could justify closer surveillance, but a specificity of this level would generate a substantial false-positive burden.

A separate deep-learning approach, trained on preoperative AS-OCT scans from 31 rebubbling and 31 non-rebubbling eyes, reported an AUC of 0.964 with a sensitivity of 96.7% and a specificity of 91.5% (26). When tested with a different surgeon and imaging environment, the same algorithm maintained solid performance but showed a decline, yielding an AUC of 0.875, a sensitivity of 78.9%, and a specificity of 78.6% (27). The model evidently learned surgical techniques and local postoperative management.

Registry data give a different view of the same problem. Among 3,647 posterior lamellar keratoplasties, detachment occurred in 9.9%; lasso regression, a decision tree, and a random forest reached independent-test AUC values of 0.70, 0.65, and 0.72, respectively (28). Moderate discrimination is expected: detachment arises from donor, recipient, surgical, and postoperative factors, several of which are simply not recorded in routine data.

The realistic near-term role, therefore, is risk-adapted follow-up rather than autonomous treatment selection. A model that guides counseling, the timing of AS-OCT, gas-fill assessment, and the date of early review would be highly useful. Any prospective implementation should be judged by delayed rebubbling, unnecessary visits, false reassurance, patient burden, and adverse events, rather than by AUC.

### 6.2 Automated detachment segmentation and mapping

Quantifying a detachment that has already occurred is significantly closer to clinical implementation than predicting a future occurrence. Heslinga et al. developed deep convolutional networks to localize detached graft tissues in AS-OCT B-scans and construct *en face* detachment maps, achieving a Dice score of 0.896 for horizontal detachment projections compared with 0.880 for a second DMEK expert—an agreement comparable to that of a second reader, at least for this specific projection task (29).

A clinically useful report should provide the detached area, maximum height, central involvement, distance from the visual axis, and serial change after image registration, alongside scan quality and uncertainty. This type of output would harmonize research definitions and replace vague descriptions such as small, peripheral, or central. It should not recommend rebubbling on its own, as that decision also depends on vision, edema, symptoms, the time since surgery, graft configuration, and the specific surgeon’s preferred criteria.

## 7 Rejection, failure, and long-term graft survival

Longitudinal endothelial morphology may signal rejection before it becomes clinically apparent. Joseph et al. analyzed postoperative specular microscopy images from 44 DMEK patients, half of whom later experienced graft rejection. Random-forest and logistic-regression models built on spatial, intensity, and shape features exceeded 80% accuracy in a held-out test set and identified rejection 1–24 months before clinical recognition (30). The findings are real and worth pursuing. However, the cohort was small and deliberately balanced, and in routine surveillance, where rejection is uncommon, positive predictive value would be considerably lower.

Analyzing long-term failure requires time-to-event methods rather than binary classification, as follow-up data are censored and risk shifts over time. In 1,335 eyes undergoing DSAEK or penetrating keratoplasty for FECD or bullous keratopathy, random survival forest-guided Cox modeling achieved a Harrell’s C-statistic of 0.701, identifying diagnosis, procedure type, recipient sex, and preoperative visual acuity as important predictors (31). In the Cornea Preservation Time Study, random survival forests analyzed 50 donor, recipient, eye-banking, and surgical variables, identifying intraoperative complications as associated with DSAEK failure (32).

A 2026 retrospective study of 114 eyes undergoing non-Descemet stripping automated endothelial keratoplasty (nDSAEK) built an XGBoost model from 10 clinical features to predict graft failure, reporting time-dependent AUC values of 0.917, 0.920, and 0.880 at 12, 24, and 36 months, respectively (33). Prioritizing the time-to-event framing and explainability analysis is the correct approach. With only 15 failure events from a single center and no external validation, the model remains strictly exploratory.

The DMEK Risk and Outcome Prediction (DROP) Score, published in 2026, combines patient, donor, surgical, and center parameters using literature-derived weights. Clinical validation involved 76 DMEK eyes and 89 controls, alongside a parallel machine-learning analysis of 6,200 *in vivo* confocal microscopy images characterizing postoperative change over time (34). The score correlated with 12-month visual acuity and ECD. Three limitations restrict the study’s conclusions: the validation was conducted at a single center, the weights were derived from literature instead of being fitted to the data, and 92.1% of the cohort fell into the moderate-risk category, which limited the ability to assess calibration, risk separation, or incremental utility. Independent external validation is required before the model can guide individual decisions.

None of this should be read independent of what we already know about long-term outcomes, namely, that graft survival and endothelial loss vary based on the procedure, indication, and donor–recipient factors (35, 36). While evidence from penetrating keratoplasty, anterior lamellar keratoplasty, or keratoconus research can guide these modeling methods, it cannot be applied directly to DMEK due to different failure mechanisms, rejection risks, and postoperative management strategies. Future models should evaluate calibration at 1, 5, and 10 years; handle competing events and repeat transplantation; and undergo revalidation whenever techniques or steroid protocols change.

## 8 Eye banks and donor tissue assessment

Eye banks maintain large image archives and structured data: donor age, ECD, death-to-preservation interval, storage time, tissue preparation, quality, and processing complications. Automated ECD measurement is an ideal candidate because it is high-volume, repetitive, and supported by an established manual reference—although that reference carries its own sampling and observer variability. Benetz et al. used 15,138 unique endothelial images from 8,169 donors and evaluated automated ECD in 1,939 test images. Their approach demonstrated substantial agreement with the manual assessment, yielding a concordance correlation coefficient of 0.77 and a bias of 123 cells/mm², with 81% of automated estimates within 10% of the manual value (37).

The safest starting point is quality control, which involves flagging discrepant counts, low-cell images, and out-of-distribution tissue for expert review. Predicting tissue-preparation failure or eventual endothelial survival is a much harder problem, given the substantial influence of recipient and surgeon factors. Donor–recipient matching raises a further question that is ethical rather than technical. An algorithm optimized for expected graft survival can quietly deprioritize complex or high-risk recipients, and allocation objectives, together with any equity constraints, must therefore remain explicit and auditable.

Representative primary studies and their current translational limitations are summarized in **Table 1**.

**Table 1:** Representative primary AI and automated image-analysis studies in corneal endothelial disease and keratoplasty.

Study/task	Dataset and independent units	Model/validation	Key result	Main limitation
Daniel et al. (9) Specular segmentation	Large real-world specular microscopy corpus; single center/device	U-Net; internal testing	Strong image-level ECD correlation; identified ungradable scans	Cell-level recall remained limited; single center/device
Sierra et al. (10) FECD morphometry	Specular microscopy images with guttae; manual morphometry reference	Signed-distance-map U-Net; manual reference	Mean ECD difference −41.9 cells/mm²	Limited external validation and device diversity
Sarin et al. (11) Automated guttae quantification	19-image validation set; 446 additional images from 109 eyes; one device	Automated image processing; expert comparison and repeatability analysis	Manual and automated GAR equivalent; repeatability ICC 0.85	Small validation set; conventional algorithm; single center/device
Vigueras-Guillén et al. (12) Post-DSAEK morphometry	383 images, 41 eyes	Dense deep network; manual comparison	98.4% successful estimates; ECD error 2.5%	One device and postoperative cohort
Heslinga et al. (14) Pachymetry	960 training B-scans/50 patients; 320 test scans	Three DL segmentation strategies; independent test	Thickness error 13.98-15.50 µm	Post-DMEK setting; device-specific
Bitton et al. (15) Edema detection	1,992 OCT images, 290 eyes	Pixel classification; postoperative thickness change reference	AUC 0.97 overall; 0.96 for FECD/normal	Detects edema, not treatment benefit
Eleiwa et al. (16) FECD staging	11,340 training images; 7,380 scans from 41 test eyes	Deep CNN; unseen scans	AUC 0.997 early and 0.974 late FECD	Many scans but few independent test eyes
Foo et al. (17) FECD detection	775 central images; 180-image external set	DL plus morphometrics; external validation	AUC fell from 0.96 internal to 0.77 external	Generalization gap across datasets
Tey et al. (18) Widefield FECD analysis	1,839 images from 155 FECD eyes; separate test set	U-Net; manual comparison	Dice 0.86 ± 0.04	Automated and manual ECD differed materially
León-Tarife et al. (24) Post-cataract EK prediction	240 eyes from 211 patients across five hospitals; 646 eye records for SSL pretraining	Hybrid CNN, SSL, ensemble learning; split testing and leave-one-center-out evaluation	Overall AUC 0.94; mean leave-one-center-out AUC approximately 0.91	Endpoint affected by referral and treatment decisions
Patefield et al. (25) DMEK detachment prediction	9,466 AS-OCT images, 74 eyes	Multiple-instance learning; held-out test	Sensitivity 92%, specificity 45%, AUC 0.63	False-positive burden; small eye-level sample
Hayashi et al. (26, 27) Rebubbling prediction	62-eye development cohort; transfer to another surgeon	CNN; cross-surgeon transfer	Development AUC 0.964; transfer AUC 0.875	Surgeon and management dependence
Muijzer et al. (28) Detachment prediction	3,647 posterior lamellar grafts	Lasso, tree, random forest; independent test	AUC 0.70, 0.65, and 0.72	Routine variables captured only moderate risk
Heslinga et al. (29) Detachment mapping	AS-OCT B-scans with expert annotations	Deep segmentation; expert comparison	Projection Dice 0.896 vs. expert 0.880	Decision threshold for rebubbling not addressed
Joseph et al. (30) Rejection prediction	44 DMEK patients; 343 pre-event images	RF/LR; held-out patient test	>0.80 accuracy; prediction 1–24 months early	Small balanced case-control cohort
Ang et al. (31) Ten-year survival	1,335 DSAEK/PK eyes	Random survival forest plus Cox model	Harrell C statistic 0.701	Mixed procedures; single regional cohort
Lin et al. (33) Graft-failure prediction after nDSAEK	114 eyes; 15 graft failures; single center	XGBoost using 10 clinical features; internal time-dependent evaluation	AUC 0.917, 0.920, and 0.880 at 12, 24, and 36 months, respectively	Few events; no external validation; nDSAEK-specific cohort
Benetz et al. (37) Donor ECD	15,138 images/8,169 donors; 1939 test images	Self-supervised and supervised segmentation	Concordance correlation 0.77; 81% within 10%	Manual counts remain the reference; image quality dependence

AI, artificial intelligence; AS-OCT, anterior-segment optical coherence tomography; AUC, area under the receiver operating characteristic curve; CNN, convolutional neural network; DL, deep learning; DMEK, Descemet membrane endothelial keratoplasty; DSAEK, Descemet stripping automated endothelial keratoplasty; ECD, endothelial cell density; EK, endothelial keratoplasty; FECD, Fuchs endothelial corneal dystrophy; GAR, guttae area ratio; ICC, intraclass correlation coefficient; LR, logistic regression; nDSAEK, non-Descemet stripping automated endothelial keratoplasty; PK, penetrating keratoplasty; RF, random forest; SSL, self-supervised learning.

## 9 Emerging applications: regenerative therapy, foundation models, and language models

The emerging applications discussed below should be interpreted in the context of the limitations identified throughout the preceding sections. Their potential value lies partly in reducing annotation burden, leveraging large unlabeled datasets, and integrating complex longitudinal information; however, they do not inherently resolve data scarcity, device dependency, dataset shift, limited external validation, or poor generalizability. Just like conventional AI, foundation models, large language models, and AI applications in regenerative therapy require the same—and, in some settings, greater—attention to multicenter validation, harmonized acquisition and outcome definitions, calibration, uncertainty, and clinically meaningful endpoints. The empirical basis is also uneven across these topics. Unlike the preceding sections, which are grounded predominantly in endothelial-specific primary studies, the evidence supporting foundation models and large language models remains largely contextual, drawing mainly from adjacent ophthalmic or broader clinical AI literature. These subsections should therefore be read as forward-looking frameworks for plausible applications rather than as evidence of established endothelial-specific clinical effectiveness.

### 9.1 Regenerative and cell-based treatments

The therapeutic landscape is transforming amid the following advancements: cell injection, tissue-engineered endothelial substitutes, Descemet stripping only, and the pharmacologic stimulation of cell migration (38). In the first clinical study in bullous keratopathy, cultured corneal endothelial cells given with a Rho-associated protein kinase inhibitor restored central ECD above 500 cells/mm² in all 11 treated eyes at 24 weeks (39). These treatments generate exactly the kind of spatial and longitudinal endpoints that reward computational analysis: regional pachymetry, posterior backscatter, cell-density distribution, optical quality, and response trajectories over time.

The evidence base has since expanded. A prospective, observational cohort study of 65 patients reported outcomes after cultured allogeneic endothelial cell transplantation, with follow-up extending up to 10 years (40). In the randomized, double-masked ESCALÓN trial, 22 eyes with bullous keratopathy or FECD received cultured endothelial cells with Y-27632; their mean central corneal thickness and visual acuity improved at 12 months with no serious treatment-emergent adverse events, although non-serious ocular events were common, including posterior capsule opacification, pupillary abnormalities or dyscoria, and transient intraocular pressure elevation (41). A first-in-human study has also shown 1-year functional recovery after transplantation of an allogeneic induced pluripotent stem cell (iPSC)-derived corneal endothelial substitute in a single eye, while underlining the stringency of genomic quality control required for such products (42).

At present, AI in regenerative endothelial therapy is an enabling technology rather than a validated basis for selecting patients. Plausible roles include automated cell-product quality control, detection of morphologic or genomic outliers, prediction of engraftment, identification of residual peripheral cell reserve, dose-response modeling, and composite spatial assessment of corneal recovery. However, because cohorts remain small and heterogeneous and the iPSC evidence relies on a single treated eye, the complex predictive models here are unusually prone to overfitting. Shared acquisition protocols, prespecified analyses, harmonized outcome definitions, and multicenter data must be prioritized, as AI-derived selection criteria would ultimately determine access to these therapies.

### 9.2 Foundation models and self-supervised pretraining

A structural weakness pervades endothelial literature: most models are trained from scratch on only a few hundred or thousand labeled images from a single device at a single center. Self-supervised pretraining on large, unlabeled collections offers a way to overcome that constraint, as demonstrated by the multicenter Scheimpflug model discussed in Section 5 (24). In retinal imaging, a foundation model pretrained on 1.6 million unlabeled images matched conventional supervised training across several downstream tasks while needing far fewer labels (43). There is no obvious reason why the same approach could not be adapted to specular microscopy, AS-OCT, and Scheimpflug data.

We identified no publicly available, externally validated foundation model built specifically for corneal endothelial imaging. Constructing one would require pooled, device-diverse, quality-annotated archives that do not currently exist as an accessible benchmark. Two caveats are worth stating plainly. Label efficiency does not remove the need for a clinically meaningful reference standard or for external validation, and a model that transfers efficiently can transfer a device-specific bias just as efficiently. General-purpose representations tend to obscure the origin of a prediction, which makes calibration, uncertainty reporting, and safe abstention more important, not less.

### 9.3 Large language models and clinical documentation

Large language models (LLMs) perform a different task from image classification. They can summarize longitudinal corneal records, extract transplant variables, draft structured operative notes, identify patients lost to follow-up, or translate a validated numerical output into a clinician-facing explanation. Their failure modes are equally distinctive: they generate probabilistic text and may fabricate facts, omit uncertainty, or cite non-existent sources. In a 2026 analysis of 200 ambient-AI draft notes across several specialties, clinicians frequently corrected factual details and added specialty-specific precision (44), which highlights the need for routine expert review rather than counting against the technology. LLMs should operate on verified data, display provenance, and require clinician approval.

A sensible future architecture should pair a validated image model with an LLM interface while keeping underlying data, such as scans, quality flags, numerical risks, and calibration information, visible. The primary risk to avoid is an LLM turning a modestly discriminative score into a high-confidence recommendation. We found no endothelial-specific clinical validation studies of an LLM. Based on current evidence, these tools belong in documentation support and research, not in corneal diagnosis or treatment selection.

## 10 Discussion

Taken as a whole, this literature has a clear pattern. The strongest results concern measurement and quality control, while the weakest involve treatment recommendations. This distinction matters in clinical practice: a segmentation model can perfectly standardize endothelial cell counts while failing to clarify if a patient requires combined surgery. The following gaps persist across the literature: limited external validation, poorly reported calibration and uncertainty, inconsistent outcome definitions, and a lack of prospective work investigating how clinicians and models perform together.

There is a pattern to where performance declines. Models perform well when comparing images from a single device against a reproducible human reference. However, they perform less well when the task becomes prognostic, the endpoint depends partly on clinician behavior, or once the model is transferred to another device, surgeon, or center. Two results illustrate this more clearly than any general argument: Foo et al. reported AUC falling from 0.96 internally to 0.77 externally (17), and Hayashi et al. reported a drop from 0.964 to 0.875 upon transfer to a different surgeon and imaging environment (26, 27). Both studies deserve credit for reporting the decline. Studies that present only internal performance should be read with these figures in mind.

### 10.1 Methodological quality and clinical readiness

#### 10.1.1 Dataset construction and reference standards

A model should be trained and tested on the population it is meant to serve, which means including ambiguous diseases, coexisting pathologies, and poor-quality images. Reference standards should be explicit and reproducible, and where the label is subjective, independent graders should use a stated adjudication rule. Every image and both eyes of a patient belong in the same data partition. External validation means a different center, device, operator, or time period; another random sample from the same archive is not external validation (45–48).

Endothelial imaging archives are fragmented across institutions and bound by strict data protection rules, making centralized pooling often unfeasible. Federated learning, in which model parameters rather than patient images move between institutions, offers one way to develop and validate across centers without transferring identifiable data (49). It is not a substitute for methodological rigor, as heterogeneity in devices, acquisition protocols, and labeling practice does not disappear, and the reference standard still has to be harmonized across sites. However, this speaks directly to the single most consistent weakness in this literature: the scarcity of device-diverse, geographically independent validation.

The unit of analysis must be stated and respected. Thousands of B-scans from a small number of eyes do not carry the same independent information as scans from thousands of patients. Confidence intervals and statistical tests should account for clustering by eye and by patient, and for postoperative outcomes, surgeon and center effects should be modeled or explicitly examined.

#### 10.1.2 Metrics, calibration, and clinical utility

Accuracy and AUC are not enough. Diagnostic studies should provide the sensitivity, specificity, and predictive values at the prevalence expected in the intended setting, along with their confidence intervals. Segmentation studies should pair Dice or intersection-over-union with errors expressed in recognizable units:recognize cells/mm², micrometers of corneal thickness, detached area, and graft height. Prognostic models need discrimination, alongside calibration plots, calibration slope and intercept, Brier score, and decision curve analysis; time-to-event models need horizon-specific calibration and proper handling of censoring.

The operating threshold should depend on the consequence. Surveillance tools can afford to favor sensitivity; however, models recommending surgery require a far higher positive predictive value and an explicit assessment of net benefit. Human-only, AI-only, and human-plus-AI performance should be compared, as the final category represents clinical reality. Ultimately, a model is useful only if it changes management in the right direction and improves outcomes, efficiency, or the patient’s experience of care.

#### 10.1.3 Robustness, uncertainty, and explainability

Performance should be reported by subgroup—disease severity, device, image quality, age, sex, ethnicity where recorded, and relevant ocular comorbidity—rather than as a single headline figure. For imaging systems, out-of-distribution detection and a safe abstention mechanism matter more than most published metrics. Saliency maps indicate which image regions a model attended to; they do not establish causality, and they are frequently over-interpreted. For structured models, clinicians often find transparent feature contributions and counterfactual examples more clinically useful. Explanations should be evaluated for stability and their ability to improve clinician understanding, rather than just how plausible they seem.

#### 10.1.4 Reporting standards

Reporting guidelines should match the study design. CLAIM 2024 covers imaging AI (50), TRIPOD+AI covers prediction-model development and evaluation (51), and the final STARD-AI guideline covers diagnostic accuracy studies involving AI (52). For trials, CONSORT-AI is used (53), and for early-stage evaluation in live clinical settings, DECIDE-AI is used (54). The FUTURE-AI consensus establishes complementary principles for trustworthy and deployable health AI (55). Naming a relevant guideline is not the same as following it; adherence should be stated explicitly and backed by a completed checklist.

The clinical maturity of the principal applications and the evidence required for further translation are summarized in **Table 2** and **Figure 2**.

**Table 2:** Clinical maturity of AI applications in corneal endothelial care according to the authors’ qualitative translational framework.

Application	Current evidence maturity	Most credible near-term role	Evidence still required
Image quality control	Early-to-moderate	Automatic acceptance/rejection and targeted manual review	Multidevice thresholds and prospective workflow testing
Endothelial morphometry	Moderate	ECD, cell area, hexagonality, and guttae quantification	Agreement across devices and advanced FECD
Edema/pachymetric mapping	Early-to-moderate	Regional and longitudinal AS-OCT quantification	Clinical thresholds linked to symptoms and outcomes
FECD diagnosis/staging	Early	Standardized grading and triage	Consecutive external cohorts and differential diagnosis
Post-cataract EK prediction	Early	Risk-informed counseling and follow-up	Absolute-risk calibration and prospective impact study
DMEK detachment mapping	Early-to-moderate	Standardized area/height/central-involvement report	Consensus action thresholds and workflow integration
Rebubbling prediction	Early	Risk-adapted early surveillance	Cross-surgeon validation and reduction in delayed treatment
Rejection/failure prediction	Early	Research alert and intensified monitoring	Large low-prevalence prospective cohorts and external validation
Eye-banking ECD assessment	Early-to-moderate	Quality control and discrepancy flagging	Operational validation and monitoring for drift
Regenerative therapy selection	Conceptual	Outcome quantification and trial phenotyping	Endothelial-specific AI models and multicenter therapeutic cohorts
Foundation models for anterior-segment imaging	Conceptual	Label-efficient pretraining for downstream endothelial tasks	Device-diverse pooled archives and downstream external validation
LLM documentation support	Conceptual	Structured summaries with source verification	Endothelial-specific prospective safety and human-factors evaluation

Ratings are qualitative application-level judgments reached by author consensus within this review. The framework was developed for interpretive clinical synthesis and is not a validated evidence-grading system; the ratings do not imply regulatory authorization, autonomous use, or demonstrated improvement in patient outcomes.

Maturity definitions: conceptual, clinically plausible use case without endothelial-specific model validation; exploratory, proof-of-concept evidence with internal validation only; early, patient-level evaluation without reliable external validation or clinical-impact evidence; early-to-moderate, clinically relevant endpoint with at least one independent, temporal, or cross-site validation; moderate, repeated validation of a measurement or workflow task with clinically interpretable error.

ECD, endothelial cell density; FECD, Fuchs endothelial corneal dystrophy; AS-OCT, anterior-segment optical coherence tomography; EK, endothelial keratoplasty; DMEK, Descemet membrane endothelial keratoplasty; LLM, large language model.

**Figure 2:**
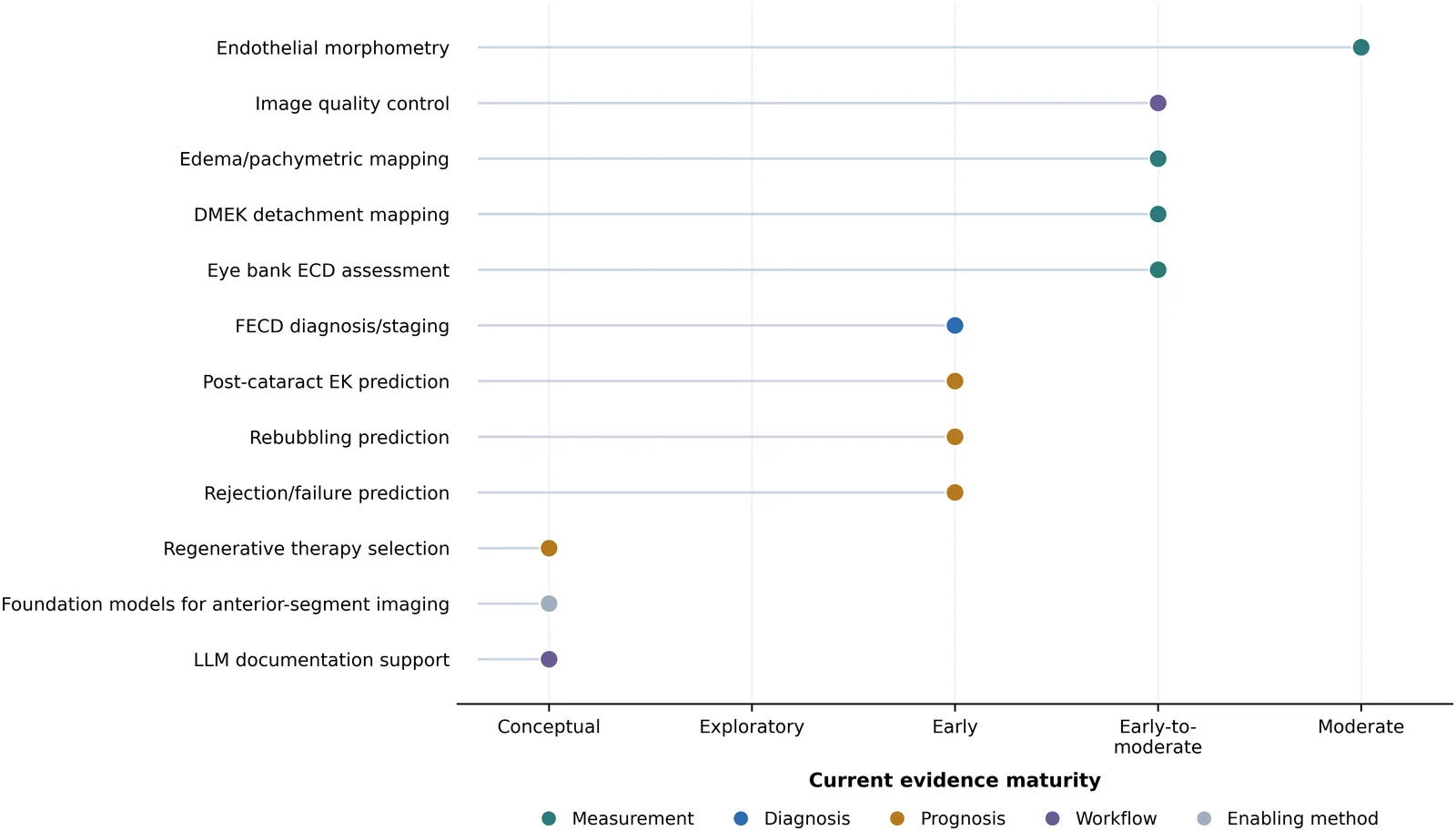
Current evidence maturity of artificial intelligence applications in corneal endothelial care. Each application is placed on the author-defined qualitative maturity scale described in Section 2 and **Table 2** and is color-coded by task type. Ratings are application-level judgments reached by author consensus and do not imply regulatory authorization, autonomous use, or demonstrated improvement in patient outcomes. AS-OCT, anterior-segment optical coherence tomography; DMEK, Descemet membrane endothelial keratoplasty; ECD, endothelial cell density; FECD, Fuchs endothelial corneal dystrophy; LLM, large language model.

### 10.2 Clinical implementation and governance

Implementation requires more than exporting a probability. The output must appear at the point of decision, integrate with the imaging software and the electronic health record, and minimize clicks and alerts; alert fatigue undermines good tools more reliably than imperfect accuracy. Institutions must assign responsibility for image quality, software updates, cybersecurity, model monitoring, user training, and adverse-event reporting. Fixed and continuously learning systems require different update controls and regulatory strategies.

Monitoring after deployment should cover calibration drift, changes in devices and acquisition protocols, shifts in the population, patterns of override, subgroup performance, and any disparity that was not anticipated. The World Health Organization’s guidance on AI for health emphasizes transparency, accountability, inclusiveness, and protection of autonomy (56). These principles stop being abstract the moment a model influences access to donor tissue, surgery, or an emerging cell therapy.

Algorithmic bias may arise when training datasets under-represent specific demographic groups, disease severities, imaging devices, referral settings, or healthcare systems. Fairness should therefore be evaluated through subgroup performance and error analyses before deployment and monitored prospectively thereafter. Without such safeguards, AI could reinforce rather than reduce existing healthcare disparities, including differences in access to specialist assessment, keratoplasty, donor tissue, and emerging regenerative therapies (46, 48, 56).

In practice, this should look like collaboration rather than delegation. The model quantifies images consistently and summarizes risk; the corneal specialist weighs symptoms, the state of the fellow eye, ocular comorbidity, surgical feasibility, patient preferences, and all factors missing from the dataset. A safe system makes it easy to open the source image, view its limitations, and disagree with the output.

The minimum requirements for clinical deployment are summarized in **Table 3**.

**Table 3:** Model-specific minimum requirements before clinical deployment.

Domain	Minimum requirement	Rationale
Intended use	Specify user, population, input, output, time horizon, and action	Prevents repurposing beyond validated scope
Data split	Patient-level separation with no fellow-eye or serial-image leakage	Avoids inflated performance
External validation	Independent center/device/operator and preferably temporal testing	Assesses generalizability
Calibration (probabilistic models)	Calibration plot, slope/intercept, and horizon-specific calibration	Makes absolute risk interpretable
Utility (decision-support models)	Decision-curve analysis or prospective clinical impact study	Shows benefit beyond AUC
Robustness	Subgroups by device, severity, quality, and relevant demographics	Identifies failure modes and inequity
Uncertainty	Quality flag, confidence estimate, and safe abstention	Supports deferral to expert review
Human factors	Human-only, AI-only, and human–AI comparison	Defines the collaborative role
Regulatory status	Device classification, conformity assessment, and documented change management for adaptive models	Aligns clinical claims and software updates with legal obligations
Monitoring	Drift, override, safety, and adverse-event surveillance	Maintains performance after deployment

Applicability note: requirements should be selected according to the intended use and model type; calibration and decision-curve analysis are primarily relevant to probabilistic diagnostic or prognostic models and may not apply to pure segmentation or image quality-control systems.

AUC, area under the receiver operating characteristic curve.

### 10.3 Regulatory and data protection context

Once these applications inform clinical decisions, they are medical device software, not research tools. In the European Union, Rule 11 of Regulation (EU) 2017/745 places software that provides information used for diagnostic or therapeutic decisions in Class IIa or above, depending on the severity of the potential consequence (57). The practical implication is proportionate: a system that recommends combined cataract surgery and DMEK carries a much heavier conformity-assessment burden than one that just flags an ungradable specular image.

Regulation (EU) 2024/1689, the Artificial Intelligence Act, adds a second layer. Under Article 6 (1), AI systems that are safety components of regulated medical devices, or that constitute such devices, are high-risk wherever the product requires third-party conformity assessment (58). The resulting obligations—risk management, data governance, technical documentation, logging, transparency, human oversight, accuracy, and robustness—map closely onto the methodological recommendations in Section 10.1, demonstrating how rigorous development and regulatory readiness are converging. The timetable is phased, and developers should verify the applicable dates and guidance at the time of deployment. Adaptive or continuously learning models require documented change management, validation, and postdeployment monitoring to ensure that an update does not silently invalidate the evidence supporting the original conformity assessment.

Finally, identifiable or pseudonymized corneal images constitute personal data concerning health under Regulation (EU) 2016/679; only truly anonymous data fall outside its scope (59). Multicenter development therefore requires a lawful basis, data minimization, and documented governance, providing a further practical argument for federated approaches. Authors reporting endothelial AI studies should state the regulatory status of the system evaluated, as a research prototype and a CE-marked device support very different clinical claims.

### 10.4 Research priorities

The field needs to move from proof-of-concept classification toward prospective decision support. The projects we would prioritize are 1) multi-device external validation of FECD segmentation and staging; 2) absolute-risk models for endothelial keratoplasty after cataract surgery, comparing phacoemulsification alone, combined DMEK, and staged treatment head to head; 3) standardized AS-OCT detachment maps tied to consensus rebubbling criteria; 4) dynamic models drawing on longitudinal ECD, pachymetry, medication exposure, and inflammatory events to anticipate rejection or failure; 5) eye-banking tools evaluated inside real operational workflows; and 6) prospective studies measuring patient outcomes, resource use, clinician behavior, and unintended consequences.

None of this is achievable without data infrastructure. Multicenter datasets should preserve device metadata, acquisition quality, laterality, repeated visits, surgeon and center identifiers, and clinically meaningful outcomes. Common data dictionaries and open benchmark subsets under transparent governance would advance the field as much as any new architecture. External validation belongs within the development process, rather than being appended as an optional final experiment.

### 10.5 Limitations of this review

This review has clear limits. We searched a single database and prioritized English-language human studies, with the aim of interpretive clinical synthesis rather than exhaustive retrieval. Performance estimates are reported as published, not recalculated or pooled, and direct comparison is limited by heterogeneity in patients, devices, units of analysis, outcomes, and validation strategy. The sections on regenerative therapy, foundation models, and language models map emerging use cases and include necessary contextual studies that did not themselves evaluate endothelial AI. The maturity categories in **Table 2** are an interpretive framework rather than a validated grading system. Accordingly, relevant studies indexed outside PubMed/MEDLINE (including engineering conference papers, non-English reports, and very recent work) may have been missed, and the absence of duplicate screening and formal risk-of-bias appraisal limits the reproducibility and formal certainty of the evidence synthesis.

## 11 Conclusion

AI can impact almost every stage of corneal endothelial care, but the quality of evidence supporting each application varies significantly. Image quality control, automated morphometry, pachymetric and edema mapping, quantification of DMEK detachment, and automated donor endothelial cell density measurement in eye banking are the closest to clinical use, as they standardize measurements we already make and can be checked directly against expert annotation. Staging FECD, predicting decompensation after cataract surgery, predicting rebubbling, flagging rejection, and modeling long-term graft survival are all promising, but none are ready yet; each needs external and prospective validation first.

Whether these tools earn a place in clinical practice will not be settled by AUC alone. It will be settled by calibration, reproducibility, net benefit, fairness, and demonstrable improvement in care. The realistic objective is not an autonomous system that replaces the corneal specialist, but one that performs the quantitative work reliably while clinical judgment remains with the surgeon.

## References

[1] Bonanno JA . Molecular mechanisms underlying the corneal endothelial pump. Exp Eye Res. (2012) 95:2–7. doi: 10.1016/j.exer.2011.06.004 21693119 PMC3199349

[2] Matthaei M Hribek A Clahsen T Bachmann B Cursiefen C Jun AS . Fuchs endothelial corneal dystrophy: clinical, genetic, pathophysiologic, and therapeutic aspects. Annu Rev Vis Sci. (2019) 5:151–75. doi: 10.1146/annurev-vision-091718-014852 31525145

[3] Woo JH Ang M Htoon HM Tan D Mehta JS . Descemet membrane endothelial keratoplasty versus Descemet stripping automated endothelial keratoplasty and penetrating keratoplasty. Am J Ophthalmol. (2019) 207:288–303. doi: 10.1016/j.ajo.2019.06.012 31228467

[4] Price FW Jr. Price MO . Evolution of endothelial keratoplasty. Cornea. (2013) 32:S28–32. doi: 10.1097/ICO.0b013e3182a0a307 24104929

[5] Liu S Kandakji L Stupnicki A Sumodhee D Leucci MT Hau S . Current applications of artificial intelligence for Fuchs endothelial corneal dystrophy: a systematic review. Transl Vis Sci Technol. (2025) 14:12. doi: 10.1167/tvst.14.6.12 40478592 PMC12155719

[6] Nguyen T Ong J Masalkhi M Waisberg E Zaman N Sarker P . Artificial intelligence in corneal diseases: a narrative review. Contact Lens Anterior Eye. (2024) 47:102284. doi: 10.1016/j.clae.2024.102284 39198101 PMC11581915

[7] Pagano L Posarelli M Giannaccare G Coco G Scorcia V Romano V . Artificial intelligence in cornea and ocular surface diseases. Saudi J Ophthalmol. (2023) 37:179–84. doi: 10.4103/sjopt.sjopt_52_23 38074299 PMC10701143

[8] Tey KY Cheong EZK Ang M . Potential applications of artificial intelligence in image analysis in cornea diseases: a review. Eye Vis (Lond). (2024) 11:10. doi: 10.1186/s40662-024-00376-3 38448961 PMC10919022

[9] Daniel MC Atzrodt L Bucher F Wacker K Böhringer S Reinhard T . Automated segmentation of the corneal endothelium in a large set of real-world specular microscopy images using the U-Net architecture. Sci Rep. (2019) 9:4752. doi: 10.1038/s41598-019-41034-2 30894636 PMC6426887

[10] Sierra JS Pineda J Rueda D Tello A Prada AM Galvis V . Corneal endothelium assessment in specular microscopy images with Fuchs dystrophy via deep regression of signed distance maps. BioMed Opt Express. (2023) 14:335–51. doi: 10.1364/BOE.477495 36698671 PMC9842012

[11] Sarin SR Abou Assi J Skeie JM Deman P Zimmerman MB McGranahan DM . Validation of automated software for guttae quantification in Fuchs endothelial corneal dystrophy. Transl Vis Sci Technol. (2026) 15:8. doi: 10.1167/tvst.15.5.8 42101417 PMC13175217

[12] Vigueras-Guillén JP van Rooij J Engel A Lemij HG van Vliet LJ Vermeer KA . Deep learning for assessing the corneal endothelium from specular microscopy images up to 1 year after ultrathin-DSAEK surgery. Transl Vis Sci Technol. (2020) 9:49. doi: 10.1167/tvst.9.2.49 32884856 PMC7445361

[13] Karaca EE Işık FD Hassanpour R Oztoprak K Kemer ÖE . Machine learning-based endothelial cell image analysis of patients undergoing Descemet membrane endothelial keratoplasty surgery. BioMed Tech (Berl). (2024) 69:481–9. doi: 10.1515/bmt-2023-0126 38491745

[14] Heslinga FG Lucassen RT van den Berg MA van der Hoek L Pluim JPW Cabrerizo J . Corneal pachymetry by AS-OCT after Descemet membrane endothelial keratoplasty. Sci Rep. (2021) 11:13976. doi: 10.1038/s41598-021-93186-9 34234179 PMC8263705

[15] Bitton K Zéboulon P Ghazal W Rizk M Elahi S Gatinel D . Deep learning model for the detection of corneal edema before Descemet membrane endothelial keratoplasty on optical coherence tomography images. Transl Vis Sci Technol. (2022) 11:19. doi: 10.1167/tvst.11.12.19 36583911 PMC9807180

[16] Eleiwa T Elsawy A Özcan E Abou Shousha MA . Automated diagnosis and staging of Fuchs endothelial cell corneal dystrophy using deep learning. Eye Vis (Lond). (2020) 7:44. doi: 10.1186/s40662-020-00209-z 32884962 PMC7460770

[17] Foo VHX Lim GYS Liu YC Ong HS Wong E Chan S . Deep learning for detection of Fuchs endothelial dystrophy from widefield specular microscopy imaging: a pilot study. Eye Vis (Lond). (2024) 11:11. doi: 10.1186/s40662-024-00378-1 38494521 PMC10946096

[18] Tey KY Lee BJH Ng C Wong QY Panda SK Dash A . Deep learning analysis of widefield cornea endothelial imaging in Fuchs dystrophy. Ophthalmol Sci. (2026) 6:100914. doi: 10.1016/j.xops.2025.100914 41140904 PMC12548096

[19] Cheong EZK Win KY Wong QY Sim R Ng CYL Htoon HM . Deep learning-assisted widefield endothelial imaging in Descemet membrane endothelial keratoplasty. Front Med (Lausanne). (2025) 12:1715673. doi: 10.3389/fmed.2025.1715673 41341834 PMC12669210

[20] Patel SV Hodge DO Treichel EJ Spiegel MR Baratz KH . Predicting the prognosis of Fuchs endothelial corneal dystrophy by using Scheimpflug tomography. Ophthalmology. (2020) 127:315–23. doi: 10.1016/j.ophtha.2019.09.033 31685256

[21] Sun SY Wacker K Baratz KH Patel SV . Determining subclinical edema in Fuchs endothelial corneal dystrophy: revised classification using Scheimpflug tomography. Ophthalmology. (2019) 126:195–204. doi: 10.1016/j.ophtha.2018.09.020 30153944

[22] Ali M Cho K Srikumaran D . Fuchs dystrophy and cataract: diagnosis, evaluation and treatment. Ophthalmol Ther. (2023) 12:691–704. doi: 10.1007/s40123-022-00637-1 36637659 PMC10011243

[23] Tourabaly M Knoeri J Georgeon C Borderie V . Review of the literature: surgery indications for Fuchs endothelial corneal dystrophy. J Clin Med. (2025) 14:2365. doi: 10.3390/jcm14072365 40217815 PMC11989955

[24] León-Tarife P Arnalich-Montiel F Mingo-Botín D Díaz de María F Stokking M Saenz-Madrazo N . Self-supervised learning and hybrid deep models for predicting the progression of Fuchs endothelial corneal dystrophy after cataract surgery. Comput Methods Programs BioMed. (2026) 273:109100. doi: 10.1016/j.cmpb.2025.109100 41106173

[25] Patefield A Meng Y Airaldi M Coco G Vaccaro S Parekh M . Deep learning using preoperative anterior-segment OCT predicts graft detachment in Descemet membrane endothelial keratoplasty. Transl Vis Sci Technol. (2023) 12:14. doi: 10.1167/tvst.12.5.14 37184500 PMC10187791

[26] Hayashi T Tabuchi H Masumoto H Morita S Oyakawa I Inoda S . A deep learning approach in rebubbling after Descemet membrane endothelial keratoplasty. Eye Contact Lens. (2020) 46:121–6. doi: 10.1097/ICL.0000000000000634 31425350

[27] Hayashi T Iliasian RM Matthaei M Schrittenlocher S Masumoto H Tanabe M . Transferability of an artificial intelligence algorithm predicting rebubblings after Descemet membrane endothelial keratoplasty. Cornea. (2023) 42:544–8. doi: 10.1097/ICO.0000000000003049 35543586

[28] Muijzer MB Hoven CMW Frank LE Vink G Wisse RPL Bartels MC . A machine learning approach to explore predictors of graft detachment following posterior lamellar keratoplasty: a nationwide registry study. Sci Rep. (2022) 12:17705. doi: 10.1038/s41598-022-22223-y 36271020 PMC9586999

[29] Heslinga FG Alberti M Pluim JPW Cabrerizo J Veta M . Quantifying graft detachment after Descemet membrane endothelial keratoplasty with deep convolutional neural networks. Transl Vis Sci Technol. (2020) 9:48. doi: 10.1167/tvst.9.2.48 32884855 PMC7445365

[30] Joseph N Benetz BA Chirra P Menegay H Oellerich S Baydoun L . Machine learning analysis of postkeratoplasty endothelial cell images for the prediction of future graft rejection. Transl Vis Sci Technol. (2023) 12:22. doi: 10.1167/tvst.12.2.22 36790821 PMC9940770

[31] Ang M He F Lang S Sabanayagam C Cheng CY Arundhati A . Machine learning to analyze factors associated with ten-year graft survival of keratoplasty for cornea endothelial disease. Front Med (Lausanne). (2022) 9:831352. doi: 10.3389/fmed.2022.831352 35721073 PMC9200960

[32] O'Brien RC Ishwaran H Szczotka-Flynn LB Lass JH Cornea Preservation Time Study Group . Random survival forests analysis of intraoperative complications as predictors of Descemet stripping automated endothelial keratoplasty graft failure in the Cornea Preservation Time Study. JAMA Ophthalmol. (2021) 139:191–7. doi: 10.1001/jamaophthalmol.2020.5743 33355637 PMC7758826

[33] Lin Y Hu Y Xie Z Fang X Luo S Xiao X . Long-term clinical outcomes of nDSAEK and machine learning-based prediction of graft survival in corneal endothelial decompensation. Clin Ophthalmol. (2026) 20:612110. doi: 10.2147/OPTH.S612110 42338499 PMC13286017

[34] Işık FD Karaca EE Oztoprak K Yumusak S Kemer OE . Development and clinical validation of the DMEK Risk and Outcome Prediction (DROP) Score: a dynamic temporal machine learning framework. J Clin Med. (2026) 15:664. doi: 10.3390/jcm15020664 41598603 PMC12841651

[35] Price MO Fairchild KM Price DA Price FW Jr . Descemet stripping endothelial keratoplasty: five-year graft survival and endothelial cell loss. Ophthalmology. (2011) 118:725–9. doi: 10.1016/j.ophtha.2010.08.012 21035862

[36] Price DA Kelley M Price FW Jr. Price MO . Five-year graft survival of Descemet membrane endothelial keratoplasty versus Descemet stripping endothelial keratoplasty and the effect of donor sex matching. Ophthalmology. (2018) 125:1508–14. doi: 10.1016/j.ophtha.2018.03.050 29731147

[37] Benetz BAM Shivade VS Joseph NM Romig NJ McCormick JC Chen J . Automatic determination of endothelial cell density from donor cornea endothelial cell images. Transl Vis Sci Technol. (2024) 13:40. doi: 10.1167/tvst.13.8.40 39177992 PMC11346145

[38] Soh YQ Mehta JS . Regenerative therapy for Fuchs endothelial corneal dystrophy. Cornea. (2018) 37:523–7. doi: 10.1097/ICO.0000000000001518 29384808

[39] Kinoshita S Koizumi N Ueno M Okumura N Imai K Tanaka H . Injection of cultured cells with a ROCK inhibitor for bullous keratopathy. N Engl J Med. (2018) 378:995–1003. doi: 10.1056/NEJMoa1712770 29539291

[40] Kinoshita S Ueno M Toda M Imai K Tomioka Y Numa K . Long-term corneal rejuvenation after transplantation of cultured human corneal endothelial cells. Ophthalmology. (2025) 132:1105–13. doi: 10.1016/j.ophtha.2025.05.020 40447112

[41] Holland EJ Yeu E Giegengack M Berdahl J Jordan E Quesada R . ESCALÓN: a prospective randomized trial of corneal endothelial cell therapy in subjects with corneal edema. Cornea. (2025) 44:1497–507. doi: 10.1097/ICO.0000000000003844 40032264

[42] Hirayama M Hatou S Nomura M Hokama R Hirayama OI Inagaki E . A first-in-human clinical study of an allogenic iPSC-derived corneal endothelial cell substitute transplantation for bullous keratopathy. Cell Rep Med. (2025) 6:101847. doi: 10.1016/j.xcrm.2024.101847 39809262 PMC11866429

[43] Zhou Y Chia MA Wagner SK Ayhan MS Williamson DJ Struyven RR . A foundation model for generalizable disease detection from retinal images. Nature. (2023) 622:156–63. doi: 10.1038/s41586-023-06555-x 37704728 PMC10550819

[44] Guo Y Hu D Yang Z Kim S Tran B Lee J . What do clinicians edit in ambient AI-drafted clinical documentation? A qualitative content analysis. J Am Med Inform Assoc. (2026) 33:1457–65. doi: 10.1093/jamia/ocag073 42118974 PMC13386006

[45] Kelly CJ Karthikesalingam A Suleyman M Corrado G King D . Key challenges for delivering clinical impact with artificial intelligence. BMC Med. (2019) 17:195. doi: 10.1186/s12916-019-1426-2 31665002 PMC6821018

[46] Wiens J Saria S Sendak M Ghassemi M Liu VX Doshi-Velez F . Do no harm: a roadmap for responsible machine learning for health care. Nat Med. (2019) 25:1337–40. doi: 10.1038/s41591-019-0548-6 31427808

[47] Nagendran M Chen Y Lovejoy CA Gordon AC Komorowski M Harvey H . Artificial intelligence versus clinicians: systematic review of design, reporting standards, and claims of deep learning studies. BMJ. (2020) 368:m689. doi: 10.1136/bmj.m689 32213531 PMC7190037

[48] Cabitza F Rasoini R Gensini GF . Unintended consequences of machine learning in medicine. JAMA. (2017) 318:517–8. doi: 10.1001/jama.2017.7797 28727867

[49] Rieke N Hancox J Li W Milletarì F Roth HR Albarqouni S . The future of digital health with federated learning. NPJ Digit Med. (2020) 3:119. doi: 10.1038/s41746-020-00323-1 33015372 PMC7490367

[50] Tejani AS Klontzas ME Gatti AA Mongan JT Moy L Park SH . Checklist for artificial intelligence in medical imaging (CLAIM): 2024 update. Radiol Artif Intell. (2024) 6:e240300. doi: 10.1148/ryai.240300 38809149 PMC11304031

[51] Collins GS Moons KGM Dhiman P Riley RD Beam AL Van Calster B . TRIPOD+AI statement: updated guidance for reporting clinical prediction models that use regression or machine learning methods. BMJ. (2024) 385:e078378. doi: 10.1136/bmj-2023-078378 38626948 PMC11019967

[52] Sounderajah V Guni A Liu X Collins GS Karthikesalingam A Markar SR . The STARD-AI reporting guideline for diagnostic accuracy studies using artificial intelligence. Nat Med. (2025) 31:3283–9. doi: 10.1038/s41591-025-03953-8 40954311

[53] Liu X Rivera SC Moher D Calvert MJ Denniston AK CONSORT-AI and SPIRIT-AI Extension Group . Reporting guidelines for clinical trial reports for interventions involving artificial intelligence: the CONSORT-AI extension. Nat Med. (2020) 26:1364–74. doi: 10.1038/s41591-020-1034-x 32908283 PMC7598943

[54] Vasey B Nagendran M Campbell B Clifton DA Collins GS Denaxas S . Reporting guideline for the early-stage clinical evaluation of decision support systems driven by artificial intelligence: DECIDE-AI. Nat Med. (2022) 28:924–33. doi: 10.1038/s41591-022-01772-9 35585198

[55] Lekadir K Frangi AF Porras AR Glocker B Cintas C Langlotz CP . FUTURE-AI: international consensus guideline for trustworthy and deployable artificial intelligence in healthcare. BMJ. (2025) 388:e081554. doi: 10.1136/bmj-2024-081554 39909534 PMC11795397

[56] World Health Organization . Ethics and Governance of Artificial Intelligence for Health. Geneva: World Health Organization (2021).

[57] European Parliament and Council of the European Union . Regulation (EU) 2017/745 on medical devices. Off J Eur Union. (2017) L117:1–175.

[58] European Parliament and Council of the European Union . Regulation (EU) 2024/1689 laying down harmonised rules on artificial intelligence (Artificial Intelligence Act). Off J Eur Union. (2024) L 2024/1689.

[59] European Parliament and Council of the European Union . Regulation (EU) 2016/679 on the protection of natural persons with regard to the processing of personal data (General Data Protection Regulation). Off J Eur Union. (2016) L119:1–88.

